# Pore‐Hierarchy Engineering of Cigarette Butt Waste Into Porous Carbon for CO_2_ Adsorption

**DOI:** 10.1002/gch2.70171

**Published:** 2026-10-24

**Authors:** Joseph F. Olorunyomi, Shinen Yu, Lison Shaju, Nandish Hosadoddi Srikantamurthy, Jianjin Ruan, Jeremy D. Landry, Xavier Mulet, Ravichandar Babarao

**Affiliations:** ^1^ School of Science RMIT University Melbourne Victoria Australia; ^2^ Centre for Advanced Materials & Industrial Chemistry (CAMIC) and ARC Centre of Excellence for Electrochemical Transformation of Carbon Dioxide School of Science RMIT University Melbourne Victoria Australia

**Keywords:** cigarette‐butt waste, CO_2_ adsorption, CO_2_/N_2_ selectivity, hierarchical pores, microporosity, waste valorization

## Abstract

Cigarette butts are a persistent plastic‐rich waste stream commonly disposed of in landfill, creating environmental and resource‐recovery challenges. Their cellulose‐acetate‐rich filters and organic components, however, provide a potential precursor for waste‐derived porous carbons. Here, whole cigarette‐butt (WCB) waste was converted into hierarchical porous carbons by carbonization followed by Na_2_CO_3_ activation. The pore structure was tuned from poorly developed porosity to a micro–mesoporous network whose development varied with Na_2_CO_3_ loading. Pore development increased across the WCB‐*x* series up to 83 wt.% Na_2_CO_3_, beyond which over‐activation reduced the effective adsorption network. WCB‐83, the highest‐uptake sample within the Na_2_CO_3_ series, combined a specific surface area of 750 m^2^ g^−^^1^ with the micropore volume of 0.24 cm^3^ g^−^^1^, delivering 3.0 mmol g^−^^1^ CO_2_ at 273 K and 1 bar. This exceeded both over‐activated WCB‐91 (2.6 mmol g^−^^1^) and the NaOH‐activated counterpart WCB‐OH‐83 (1.2 mmol g^−^^1^). At 298 K, WCB‐83 adsorbed 0.3 mmol g^−^^1^ CO_2_ at 0.15 bar and 1.0 mmol g^−^^1^ at 1 bar. Temperature‐dependent adsorption gave apparent isosteric heats of approximately 53 to 59 kJ mol^−^^1^, while Ideal Adsorbed Solution Theory (IAST) ‐predicted CO_2_/N_2_ selectivity reached 120. This work demonstrates a waste‐valorization strategy for converting cigarette‐butt waste into CO_2_‐selective hierarchical carbon adsorbents.

## Graphical abstract

Whole cigarette‐butt waste is converted into porous carbon by carbonization and Na_2_CO_3_ activation. Varying Na_2_CO_3_ loading regulates pore development and CO_2_ uptake, with WCB‐83 adsorbing 3.0 mmol g^−^^1^ at 273 K and 1 bar. The study links waste valorization with pore‐structure control in carbon adsorbents.

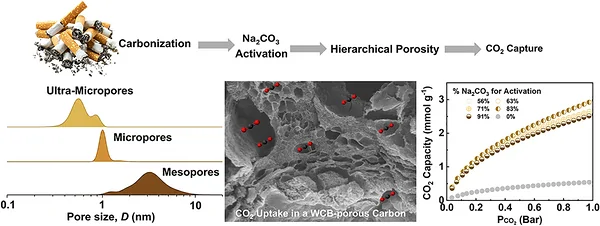

## 1 Introduction

The design of carbon‐capture materials increasingly intersects with the broader need to develop circular pathways for persistent carbon‐rich wastes. Porous solid adsorbents are widely investigated as alternatives to liquid amine absorption because they can offer lower regeneration requirements, improved operational simplicity, and greater chemical stability under suitable conditions [1, 2, 3]. Among these are porous carbons that are particularly attractive because their pore structure, surface chemistry, and adsorption behavior can be tuned through precursor selection, carbonization, and activation [4, 5]. However, the development of porous carbon sorbents should not be assessed only by adsorption capacity. The sustainability and practical relevance of the precursor, the severity of the activation route, and the relationship between pore architecture and CO_2_ uptake are equally important [6, 7, 8, 9].

The development of waste‐derived porous carbons for CO_2_ capture aligns with waste to resource strategies that convert low value or hazardous residues into functional adsorbents. Municipal and organic waste streams are increasingly recognized as accessible precursors for producing carbon materials with tunable porosity and surface chemistry, making them attractive platforms for environmental gas adsorption [10, 11, 12, 13]. This approach is especially relevant where the waste stream is abundant, difficult to remediate, and intrinsically carbon‐rich. Cigarette butt waste meets these criteria. Discarded cigarette butts are widely dispersed environmental pollutants composed of cellulose acetate filters, wrapping paper, residual tobacco, additives, ash‐forming components, heavy metals, and adsorbed organic contaminants [14, 15, 16, 17]. Their small size, persistence, and chemical complexity make them difficult to collect, separate, and manage effectively. Conventional disposal routes such as landfilling or incineration do not recover the embedded carbon value of this waste and may introduce additional environmental concerns [16, 18]. Converting cigarette butt waste into porous carbon sorbents therefore offers a route to upcycle a persistent pollutant into a higher‐value material for CO_2_ capture.

The use of cigarette butt waste as a carbon precursor is not, by itself, new. Previous studies have demonstrated cigarette‐butt‐derived carbons for hydrogen storage, electrochemical energy storage, water remediation, and adsorption of environmental contaminants [19, 20, 21]. More recent studies have also reported cigarette‐butt‐derived porous biochars for CO_2_ capture, often using hydrothermal pretreatment, nitrogen doping, mixed salts, or strong alkali activation to generate ultra micropore‐rich carbon frameworks [22]. These studies establish cigarette butt waste as a viable carbon capture resource, but they also show that the field has moved beyond simple waste‐to‐carbon conversion. A more important question is how the heterogeneous composition of whole cigarette butt waste can be used in the controlled formation of CO_2_‐accessible pore networks.

This question is central because CO_2_ adsorption in porous carbons is governed by pore architecture as well as surface chemistry. Narrow micropores and ultra‐micropores provide strong adsorption potentials for CO_2_ molecules at low pressure, but a pore network dominated only by narrow micropores can limit molecular accessibility and slow transport [23, 24]. Mesopores and larger voids do not usually provide the strongest adsorption sites, but they can improve diffusion and expose internal microporous domains. Hierarchical porosity can combine adsorption within narrow pores with access through larger pores [25, 26, 27, 28, 29].

Achieving hierarchical porosity in waste‐derived carbons is challenging because pore formation is controlled by the coupled effects of precursor composition, thermal decomposition, activator chemistry, and removal of inorganic residues during post‐treatment. This challenge is particularly relevant for whole cigarette butt waste, which more closely represents collected cigarette waste than separated filter‐only precursors. While isolated filters simplify synthesis, they do not capture the heterogeneous composition of the real waste stream, which includes cellulose acetate, paper, residual tobacco, additives, ash‐forming components, and inorganic residues. Retaining the whole cigarette butt precursor therefore provides a more practical basis for investigating how activation chemistry transforms a complex composite waste into porous carbon.

In this work, we develop a template‐free synthesis strategy for engineering hierarchical porosity in carbon derived from whole cigarette butts through Na_2_CO_3_ activation. Although Na_2_CO_3_ is an established mild activating agent for biomass carbons [30, 31], its use to engineer pore hierarchy in a composite, plastic rich waste such as whole cigarette butts remains underexplored. Here, carbonate activation regulates structural opening and the development of micropores and mesopores within the waste char, clarifying how milder activation builds a balanced pore architecture that favors CO_2_ adsorption compared with more aggressive routes. The novelty is combining a whole cigarette butt precursor from real collected waste with mild Na_2_CO_3_ activation that replaces corrosive NaOH and tunes the pore hierarchy through activator loading. Together these give a carbon whose ultra‐micropores drive its CO_2_/N_2_ selectivity.

## 2 Results and Discussion

### 2.1 Synthesis and Thermal Analysis of the Precursors

Discarded cigarette butts were converted into porous carbon through a two‐step pyrolysis–activation process as shown in Scheme 1.

**SCHEME 1:**
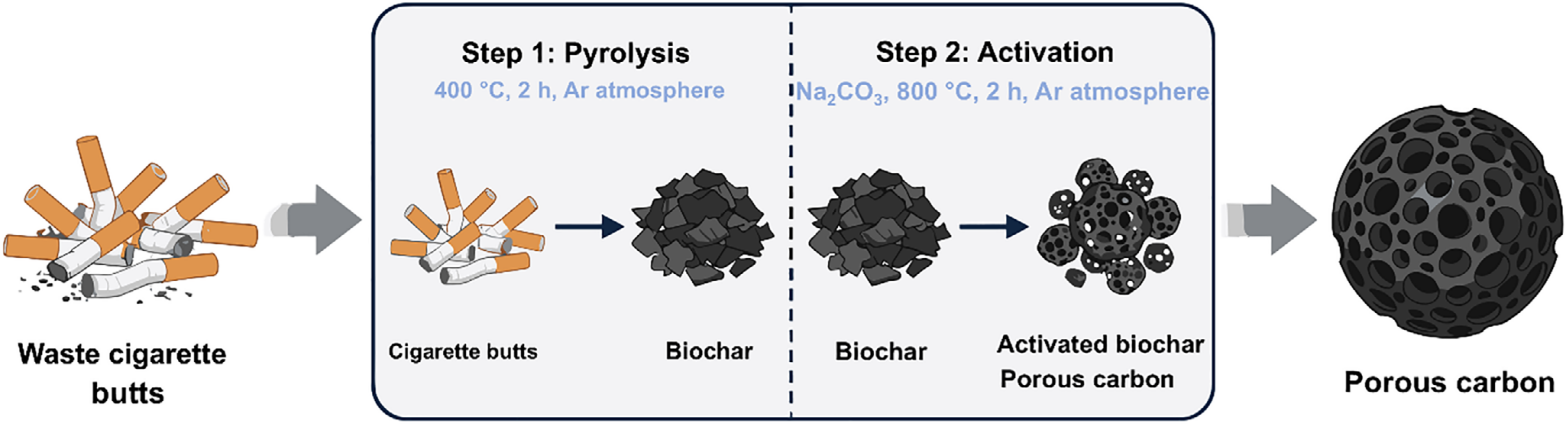
Schematic of porous carbon production from waste cigarette butts via pyrolysis and Na_2_CO_3_‐assisted activation under Ar.

This waste was processed as two precursor fractions: whole cigarette butts (WCB) and filter‐only cigarette butts (FCB). WCB contained the cellulose acetate filter together with wrapping paper and associated residues, whereas FCB consisted mainly of cellulose acetate filters after removal of the external wrapping paper. Both fractions were washed to remove sand, dust, and loosely attached contaminants, dried under vacuum, and pyrolyzed under argon at 400°C for 2 h to produce carbonaceous char intermediates. This carbonization step converted the polymer‐rich waste into a carbon‐rich solid suitable for subsequent chemical activation. The precursor composition had a clear effect on char formation. Based on thermogravimetry analysis (TGA) performed in N_2_ environment, WCB produced a substantially higher residual yield at 400°C than FCB, approximately 32% compared with 16% (Figure S1a), and the TGA‐derived yields were consistent with those obtained from bulk tube‐furnace pyrolysis. The higher yield from WCB is attributed to its more complex composition, including cellulose acetate, wrapping paper, additives, ash‐forming components, and possible trace mineral residues. Since WCB also avoids the additional separation step required to isolate filter‐only material and more closely represents actual cigarette butt waste treatment, it was selected as the main precursor for subsequent activation. Further details of the precursor thermal behavior are provided in Section S1 and Figure S1. The WCB‐derived char was then chemically activated with Na_2_CO_3_ at 800°C for 2 h under argon to develop accessible porosity. The char‐to‐Na_2_CO_3_ mass ratio was varied from 4:5 to 1:10, corresponding to Na_2_CO_3_ contents of approximately 56–91 wt.%, to examine the effect of activator loading on char transformation and pore development. Samples are labelled WCB‐*x*, in which WCB refers to the whole cigarette butt precursor and *x* is the Na_2_CO_3_ loading (wt.%) used during activation; WCB‐0 is the control carbon prepared without Na_2_CO_3_. The relatively high Na_2_CO_3_ contents were used to promote intimate contact between the salt and the carbon matrix during thermal treatment. Since pore development depends strongly on the extent of contact between Na_2_CO_3_ and carbon matrix, insufficient loading may limit chemical etching and restrict the development of accessible porosity. Similar trends have been reported for chemically activated biomass‐derived carbons, where the precursor‐to‐activating agent ratio strongly influences surface area, pore volume, and sorption performance [32]. Thermal analysis of the char–Na_2_CO_3_ mixtures confirmed that Na_2_CO_3_ was largely stable under the activation conditions but modified the high‐temperature transformation of the carbonized char (Figure S1b,c). Therefore, pore development is attributed to carbonate‐assisted char transformation, involving interfacial Na_2_CO_3_–carbon reactions, gasification of reactive carbon sites, formation of sodium‐containing intermediates, and restructuring of the carbon matrix, followed by removal of soluble inorganic species during washing. The activation temperature was held constant at 800°C across the WCB‐*x* series to isolate the effect of Na_2_CO_3_ loading on pore development. This value lies above the carbonate‐assisted char‐transformation features observed by TGA/DTG (Figure S1c) and within the temperature range established for Na_2_CO_3_ activation of biomass‐derived carbons [33, 34]. A comparison with NaOH activation showed that NaOH induced earlier and more extensive char transformation than Na_2_CO_3_, as discussed in the Supporting Information and Figure S1d,e. Figure S1f shows that the Na_2_CO_3_‐activated WCB‐*x* samples are thermally stable, retaining approximately 87%–90% of their mass at 800°C. This limited weight loss of about 10%–13% contrasts with the non‐activated WCB‐0, which shows greater degradation and lower residual mass, indicating that activation produces a more stable carbon framework.

### 2.2 Characterization of WCB‐*x* Porous Carbons

The structural consequences of Na_2_CO_3_ activation were evident from scanning electron microscopy (SEM) images shown in Figure 1. The WCB‐0 carbon annealed at 800°C (Figure 1a) showed a compact, agglomerated morphology with rounded carbonaceous domains and limited visible surface porosity. Following Na_2_CO_3_ activation, washing with deionized water, and pH adjustment, the carbon morphology became markedly more open, showing roughened and etched surfaces alongside cavities and larger open voids. At lower Na_2_CO_3_ contents (56–83 wt.%, Figure 1b–e), the activated carbons displayed localized etching and irregular pore openings, whereas higher Na_2_CO_3_ loadings (for WCB‐91, Figure 1f) produced fractured sheet‐like domains, larger cavities, and more pronounced lamellar features. The WCB‐91 carbon sample showed the most open morphology, with macropores and thinner carbon walls, indicating extensive salt‐assisted disruption of the carbon matrix.

**FIGURE 1:**
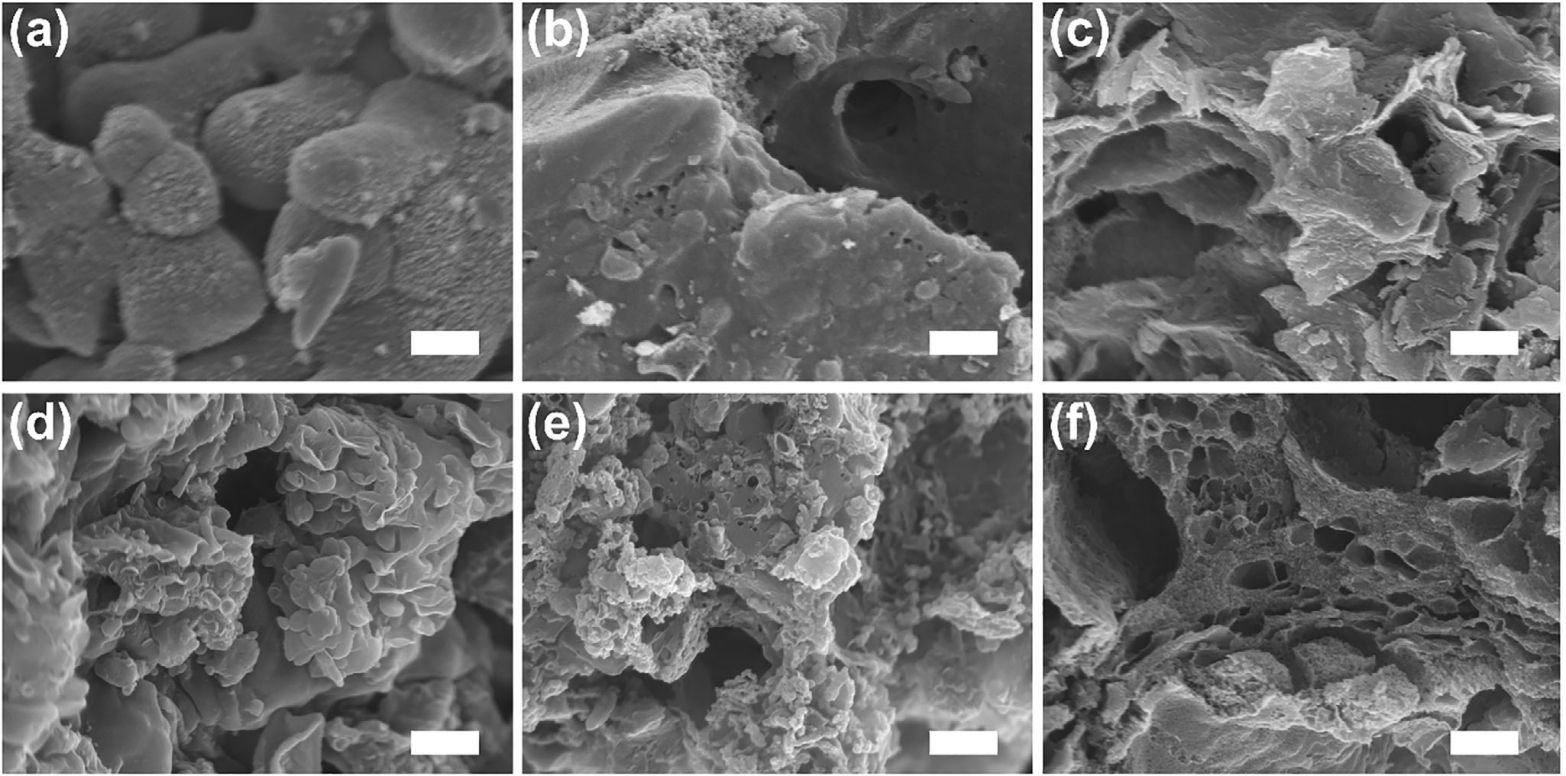
SEM images showing the morphological evolution of WCB‐derived carbons before and after Na_2_CO_3_ activation. (a) Non‐activated WCB‐derived carbon, WCB‐0; and Na_2_CO_3_‐activated and washed WCB‐derived porous carbons: (b) WCB‐56, (c) WCB‐63, (d) WCB‐71, (e) WCB‐83, and (f) WCB‐91. Scale bars: 500 nm.

Raman spectroscopy shows that all WCB‐*x* carbons retain a predominantly disordered framework rich in sp^2^ carbon after carbonization and activation (Figure 2a). The prominent D and G bands at approximately 1350 and 1580 cm^−^^1^, respectively, with (*I_D_/I_G_*) values close to unity, are characteristic of a defective sp^2^ carbon composed of small aromatic domains with abundant edge defects and limited long range graphitic ordering [35, 36, 37]. This defective signature is already present in WCB‐0, suggesting that the disorder originates from carbonization of the cigarette butt precursor rather than from Na_2_CO_3_ activation. The (*I_D_/I_G_*) ratios remain broadly unchanged across the WCB‐*x* series, so increasing Na_2_CO_3_ loading does not substantially alter the degree of carbon ordering. Activation therefore develops porosity predominantly by etching the carbon matrix, while the underlying sp^2^ framework is largely preserved.

**FIGURE 2:**
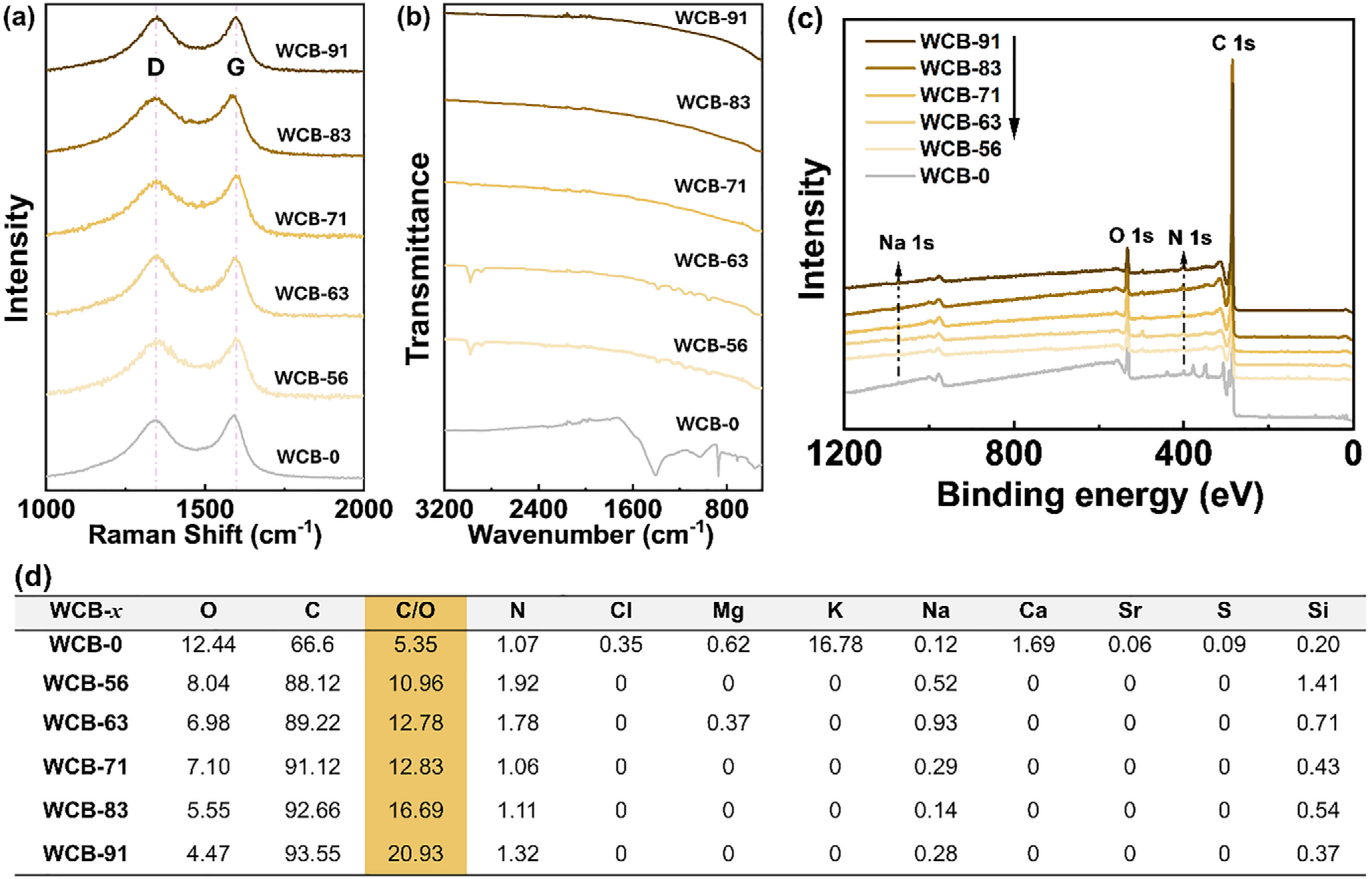
(a) Raman spectra showing the characteristic D and G bands of disordered sp^2^ carbon. (b) FTIR spectra showing weak residual surface vibrational features after thermal treatment and activation. (c) XPS survey spectra identifying the principal surface elements. (d) Table of XPS‐derived atomic compositions and C/O ratios of the WCB‐x samples.

The Fourier‐transform infrared (FTIR) and the x‐ray Photoelectron spectra (XPS) survey spectra provide complementary information on the surface chemistry of the WCB‐*x* carbons. The activated samples exhibit generally weak FTIR absorption, consistent with the extensive loss of infrared‐active surface functionalities. Weak features near 2990 and 2880 cm^−^^1^ are observed mainly for WCB‐56 and WCB‐63; although these occur within the aliphatic C─H stretching region, their low intensity and absence from the more strongly activated samples suggest that they may arise from trace adventitious surface contamination. The low‐intensity bands between approximately 1390 and 950 cm^−^^1^ are tentatively associated with overlapping vibrations from oxygenated carbon sites and residual inorganic components. (Figure 2b). These features became less pronounced in the more strongly activated samples, consistent with progressive removal or transformation of surface species during activation and washing. XPS analysis further supports this surface evolution (Figure 2c,d). Although the measured atomic concentrations represent the near‐surface composition, they enable comparison of surface elemental changes across the WCB‐*x* series. Relative to WCB‐0, which contained 66.6 at.% C and 12.44 at.% O, the activated carbons contained 88.12–93.55 at.% C and 4.47–8.04 at.% O, increasing the surface C/O ratio from approximately 5.4 to 11.0–20.9, consistent with deoxygenation and enrichment of the carbonaceous surface. Activation and acid washing also removed most of the K, Ca, Mg, Cl, Sr, and S species detected in WCB‐0, producing substantially more carbon‐rich and demineralised surfaces. Residual Na and Si contents were low (0.14–0.93 and 0.20–1.41 at.%, respectively) and showed no systematic dependence on Na_2_CO_3_ loading, consistent with effective removal of most Na‐containing species and a heterogeneous distribution of trace Si‐containing mineral residues.

Nitrogen sorption analysis at 77 K was performed to quantify the porous properties of the activated WCB‐derived carbons. The adsorption–desorption isotherms indicated in Figure 3a show a sharp N_2_ uptake at low relative pressure, confirming the presence of micropores. In addition, the hysteresis loop at intermediate‐to‐high relative pressure is consistent with an H4‐type isotherm, which is commonly associated with slit‐like mesopores in carbonaceous or disordered porous materials [38, 39]. These features indicate that Na_2_CO_3_ activation generates a micro–mesoporous carbon structure. The non‐activated carbon showed negligible N_2_ uptake, indicating that carbonization alone produced limited accessible porosity. In contrast, the activated WCB‐*x* samples exhibited distinctly higher N_2_ adsorption, confirming the role of Na_2_CO_3_ in generating organized pore hierarchy. The Brunauer–Emmett–Teller (BET) surface area increased with Na_2_CO_3_ activation level, rising from approximately 590 m^2^ g^−^^1^ for WCB‐56 to a maximum of approximately 750 m^2^ g^−^^1^ for WCB‐83, before decreasing for WCB‐91 (Figure 3b). This trend closely follows the evolution of micropore volume, which increased from 0.18 cm^3^ g^−^^1^ for WCB‐56 to 0.24 cm^3^ g^−^^1^ for WCB‐83, then decreased to 0.19 cm^3^ g^−^^1^ for WCB‐91 (Figure 3c). This correlation suggests that micropore development is the primary contributor to the enhanced surface area, while excessive Na_2_CO_3_ loading leads to loss of effective microporosity, likely through pore widening, over‐etching, or partial degradation of the porous carbon network. The mesopore volume showed a non‐monotonic trend, decreasing from 0.22 cm^3^ g^−^^1^ for WCB‐56 to 0.16 cm^3^ g^−^^1^ for WCB‐63, then increasing to a maximum of 0.28 cm^3^ g^−^^1^ for WCB‐83 before declining to 0.19 cm^3^ g^−^^1^ for WCB‐91 (Figure 3c). The simultaneous maxima in BET surface area, micropore volume, and mesopore volume for WCB‐83 indicate an optimal activation condition. Notably, the mesopore volume of WCB‐83 was approximately 17% higher than its micropore volume, suggesting a well‐balanced micro–mesoporous structure that combines high surface area with improved pore accessibility. The pore‐size distribution was analyzed to further define the textural basis of the hierarchical porosity in the activated WCB‐*x* samples. Micropore‐size distributions derived from the N_2_ isotherms using the Tarazona non‐local density functional theory (NLDFT) model [40, 41] show that the WCB‐*x* samples possess a dominant narrow micropore population centered at approximately 0.95 nm (Figure 3d). However, since N_2_ adsorption at cryogenic temperature poorly access or resolve very narrow micropores because of kinetic restrictions, CO_2_ adsorption at 273 K was used as a complementary probe for ultra microporosity [42]. The CO_2_‐derived distribution shown in Figure 3e indicate additional features at approximately 0.56 and 0.82 nm, confirming the presence of ultra‐micropores within the WCB‐*x* carbon framework. In parallel, the Barrett–Joyner–Halenda (BJH) method indicates a mesopore contribution near 4.0 nm (Figure 3f). Because BJH is known to underestimate pore sizes in carbon mesopores [43, 44], it is used here only as a qualitative indicator; the quantitative pore structure rests on the NLDFT and CO_2_ DFT analyses. The coexistence of these narrow micropores and small mesopores confirms that Na_2_CO_3_ activation generates a micro–mesoporous hierarchical structure, in which the micropores contribute substantially to internal surface area while the mesopores provide wider pore channels within the carbon framework.

**FIGURE 3:**
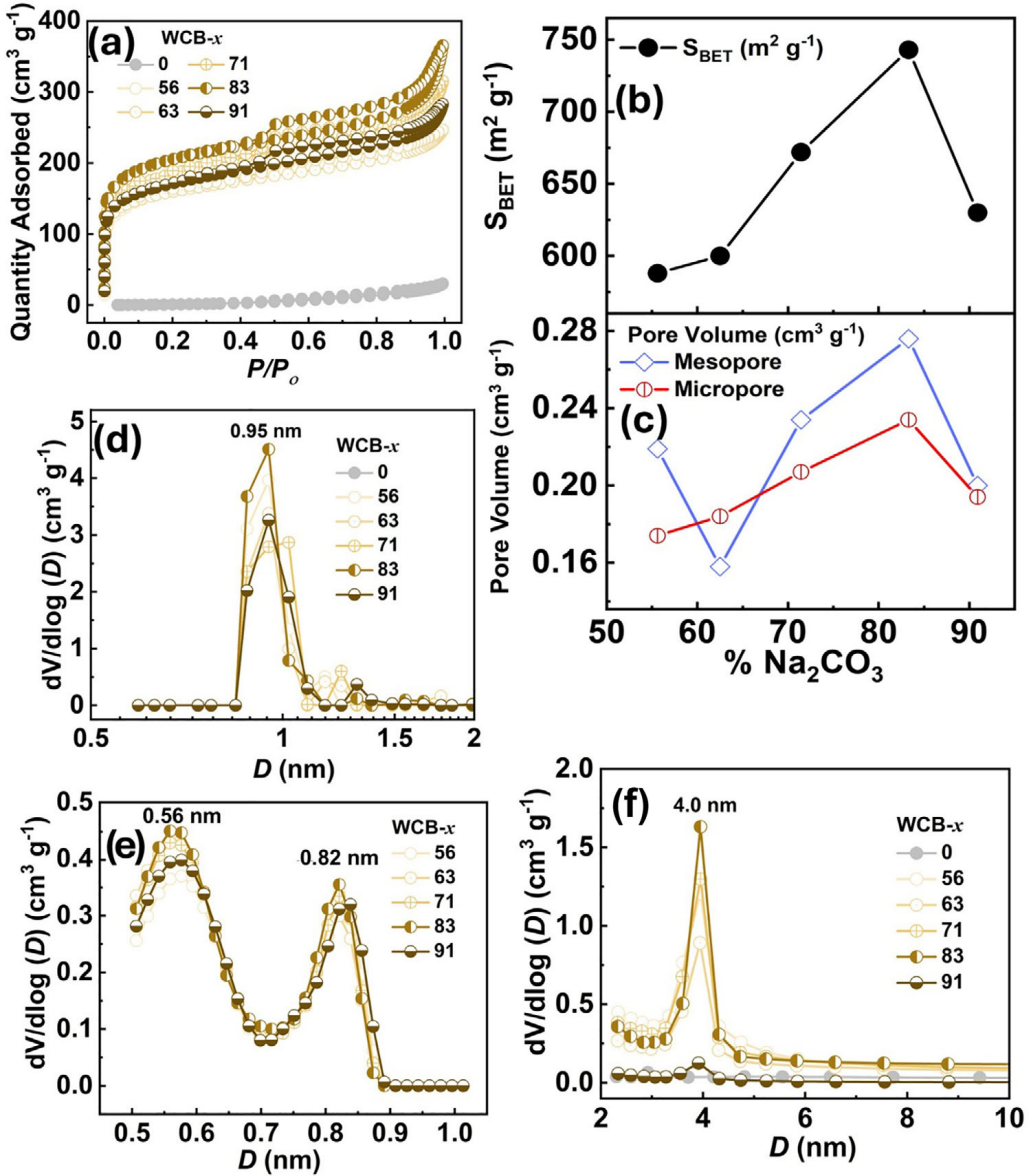
Porous properties of WCB‐x carbons, where x denotes the Na_2_CO_3_ content used during activation (x = 0, 56, 63, 71, 83, and 91 wt.%). (a) N_2_ adsorption–desorption isotherms measured at 77 K, (b) BET specific surface area, (c) micro‐ and mesopore volumes, (d) NLDFT pore‐size distribution in the micropore region, (e) CO_2_‐derived pore size distribution showing the ultra‐micropores, and (f) Barrett–Joyner–Halenda (BJH) pore‐size distribution in the mesopore region. The NLDFT pore‐size distribution of the non‐activated WCB‐0 sample is not shown because of its negligible porosity.

This hierarchical pore structure is crucial for the adsorption of CO_2_ molecules, which shall be discussed shortly. Micropores provide high surface area and strong adsorption potentials for CO_2_ molecules, whereas mesopores improve diffusion and accessibility to internal adsorption sites. The larger macropores present in the WCB‐*x* samples will further facilitate gas transport by acting as external diffusion channels into the internal micro–mesoporous network. Raman spectroscopy was used to provide structural context for the differences in porosity observed from the N_2_ sorption analyses. The N_2_ sorption data already show that WCB‐83 possesses a more balanced micro–mesoporous structure, whereas the NaOH‐activated counterpart, WCB‐OH‐83, is more predominantly microporous (Figure S2). Consistent with this difference, WCB‐83 exhibits substantially broader D and G bands, with FWHM values of 236 and 108 cm^−^^1^, respectively, compared with 110 and 60 cm^−^^1^ for WCB‐OH‐83 (Figure S3). The broader D band indicates a wider distribution of defect‐ and edge‐related sp^2^ carbon environments, while the broader G band suggests greater heterogeneity within the aromatic carbon framework. These features are consistent with carbonate‐assisted opening and fragmentation of the carbon matrix, leading to structurally diverse pore walls associated with mesopore development [45, 46]. In contrast, the sharper Raman features of WCB‐OH‐83 suggest a comparatively more uniform or selectively etched residual carbon framework, consistent with its lower mesopore contribution under the same activation loading. Thus, while Raman spectroscopy does not directly quantify pore size, it supports the structural origin of the micro–mesoporous hierarchy identified by N_2_ sorption.

### 2.3 CO_2_ Adsorption Study of WCB‐*x* Porous Carbons

The CO_2_ adsorption performance of the WCB‐derived carbons is shown in Figure 4. At 273 K and 1 bar, the CO_2_ uptake increased with Na_2_CO_3_ loading from WCB‐56 to WCB‐83, reaching a maximum of approximately 3.0 mmol g^−^^1^ for WCB‐83, before decreasing to approximately 2.6 mmol g^−^^1^ for WCB‐91 (Figure 4a,b). This trend follows the pore‐structure evolution earlier observed from N_2_ sorption, where WCB‐83 showed the most favorable combination of surface area, micropore volume, and mesopore contribution. The lower uptake of WCB‐91 suggests that excessive activation reduces the effective CO_2_ adsorption network, most likely through micropore widening, loss of high‐affinity adsorption volume, or partial structural degradation despite the more open morphology.

**FIGURE 4:**
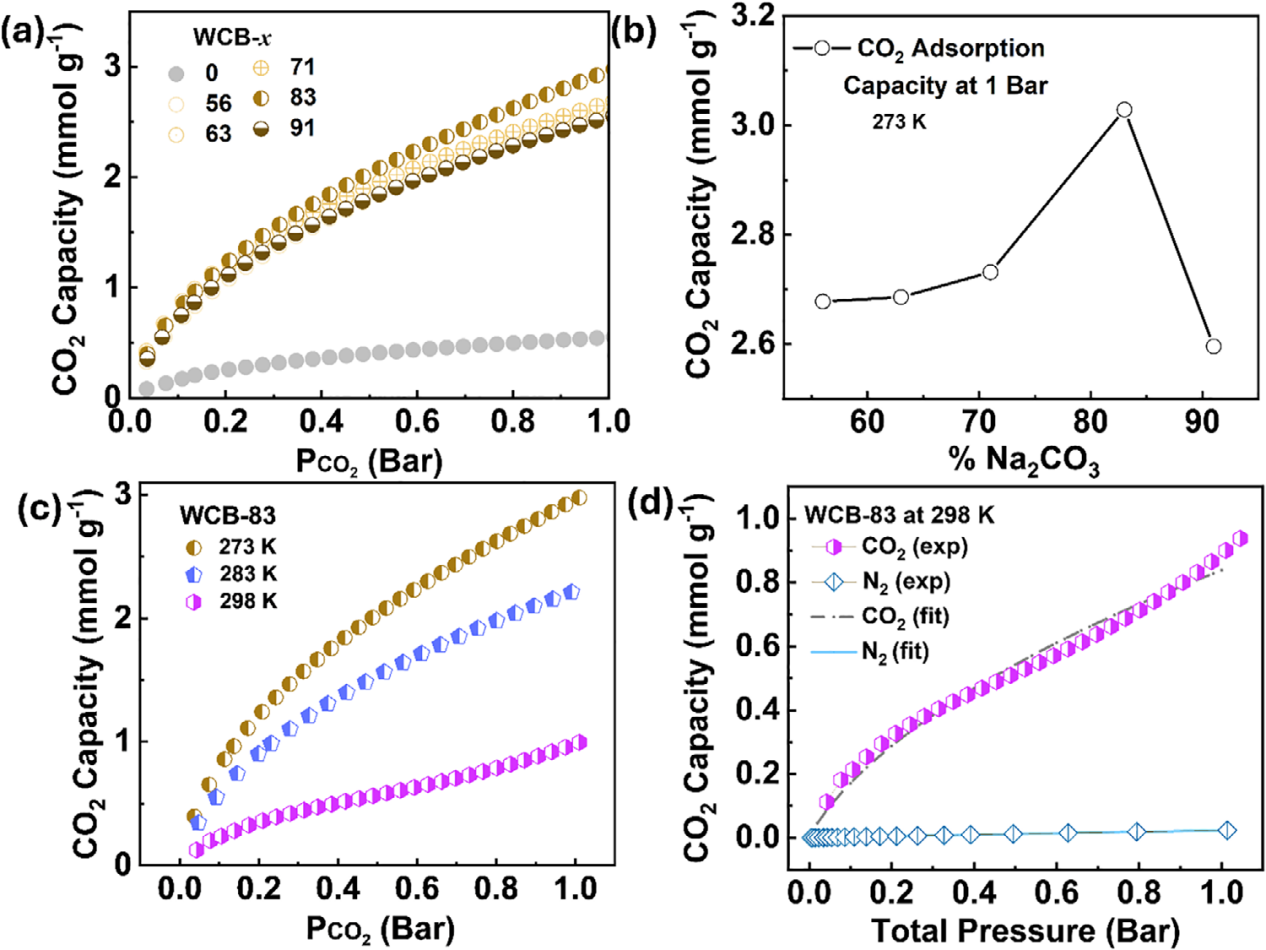
(a) CO_2_ adsorption isotherms of WCB‐*x* carbons, where *x* denotes the Na_2_CO_3_ content used during activation (*x* = 0, 56, 63, 71, 83, and 91 wt.%) at 273 K up to 1 bar. (c) CO_2_ adsorption isotherms of WCB‐83 at 273, 283 and 298 K; and (d) CO_2_ and N_2_ adsorption isotherms of WCB‐83 at 298 K with Toth model fits.

Within the Na_2_CO_3_ series, WCB‐83 exhibited the highest CO_2_ uptake, coinciding with the maximum BET surface area and micropore volume. In this structure, micropores and ultra‐micropores provide the main high‐affinity CO_2_ adsorption domains [47, 48, 49, 50], while mesopores and macropore‐like voids improve access to the internal pore network. This is consistent with the established role of narrow micropores in low‐pressure CO_2_ adsorption, where overlapping pore‐wall potentials strengthen CO_2_ confinement, whereas larger pores mainly support diffusion and transport [48, 51]. Similarly, recent work on cigarette‐butt‐derived N‐doped biochars confirms that ultra‐microporosity dominates equilibrium CO_2_ uptake, whereas larger pores mainly facilitate gas transfer through the carbon framework [6, 22]. In the present WCB‐*x* carbons, by contrast, surface nitrogen remained low across the series (1.0–1.9 at.%; Figure 2d) and did not track CO_2_ uptake: the nitrogen richest WCB‐56 (1.92 at.%) was not the best adsorbent, and WCB‐0 and WCB‐83, with nearly identical nitrogen (1.07 and 1.11 at.%), differed sixfold in uptake (≈0.5 versus 3.0 mmol g^−^^1^). The CO_2_ affinity is therefore attributed to micro and ultra‐ micropore confinement. A single NaOH control is used to isolate the effect of activation chemistry. At the same 5:1 ratio, the more aggressive NaOH gave WCB‐OH‐83 a higher BET surface area than the milder Na_2_CO_3_ gave WCB‐83 (900 versus 750 m^2^ g^−^^1^; Figure S2), yet WCB‐OH‐83 took up less than half the CO_2_ (1.2 versus ∼3.0 mmol g^−^^1^ at 273 K and 1 bar; Figure S4), showing that the difference arises from pore architecture. Beyond this single comparison, the loading dependent conclusions of this study are restricted to the consistently prepared WCB‐*x* (Na_2_CO_3_) series.

The temperature dependence of adsorption gives the isosteric heat (*Q*_st_), a direct measure of how strongly CO_2_ is bound. CO_2_ uptake decreases across the three isotherms measured at 273, 283 and 298 K, consistent with exothermic adsorption (Figure 4c). The apparent *Q_st_*, obtained through the Clausius–Clapeyron relation by interpolating *P* at fixed loading and regressing ln *P* against 1/*T*, is approximately 53 to 59 kJ mol^−^^1^ across the 0.4 to 1.0 mmol g^−^^1^ loading range (Figure S5). Each value is fitted from only three temperatures and carries a regression standard error of order 10 to 14 kJ mol^−^^1^, so the shallow maximum near 0.6 mmol g^−^^1^ falls within the scatter; the profile is read only as qualitative evidence of energetically heterogeneous adsorption [51, 52]. This magnitude reflects strong physisorption in the exceptionally narrow ultra‐micropores of WCB‐83 (≈0.56 nm, the smallest among the compared materials), where overlapping pore wall potentials intensify CO_2_ confinement [53, 54]. The activation route introduces no nitrogen or amine functionality, and the CO_2_ isotherms overlap closely over five cycles at 273 K, with the 1 bar capacity fully recovered on every cycle (≈2.8 to 3.1 mmol g^−^^1^, no systematic decline; Figure S6). Complete capacity recovery on every cycle marks the uptake as reversible physisorption; chemisorbed CO_2_ would instead accumulate and fade the capacity. The isosteric heat, near 50 kJ mol^−^^1^, is below the ∼70 to 100 kJ mol^−^^1^ typical of amine CO_2_ chemisorbents [55, 56, 57, 58], indicating a lower regeneration penalty. WCB‐83 nonetheless retains 5.55 at.% surface oxygen, so a minor contribution from polar oxygen functionalities or residual inorganic species cannot be excluded.

The CO_2_/N_2_ adsorption behavior of WCB‐83 at 298 K further demonstrates its preferential affinity for CO_2_ (Figure 4d). CO_2_ uptake increased strongly with pressure, whereas N_2_ adsorption remained much lower across the measured range. This difference reflects the stronger quadrupole interaction and greater polarizability of CO_2_, together with strong confinement within the microporous structure. To estimate binary separation performance, the single‐component CO_2_ and N_2_ isotherms were fitted using the Toth model prior to IAST calculation (as detailed in the Supporting Information). The Toth model was adopted because it better accommodates adsorption‐site heterogeneity in hierarchical porous carbons than a simple Langmuir model [59, 60, 61].

IAST predicted a CO_2_/N_2_ selectivity of approximately 110–120 at 298 K for a dry 15:85 mixture over 0.02–1.0 bar using the Toth fit, which best reproduces the measured isotherms (Figure 4f); a single site Langmuir fit gives a lower range of 64–80. These are equilibrium estimates from fitted single‐component isotherms, and the value depends on model choice, the saturation‐capacity constraint, and extrapolation beyond the measured range (fit residuals and sensitivity analyses, Figure S7).

The slight decrease at low pressure is consistent with progressive occupation of the strongest CO_2_ adsorption sites, while the sustained high selectivity at higher pressure indicates that WCB‐83 retains strong preferential CO_2_ adsorption under idealized flue‐gas‐relevant conditions.

Although the CO_2_ uptake of WCB‐83 (3.0 mmol g^−^^1^ at 273 K, 1 bar) is modest relative to several adsorbents in Table S1, it is obtained from a real waste precursor by a mild Na_2_CO_3_ activation route, and its engineered ultra‐microporosity delivers favorable CO_2_/N_2_ selectivity and a relatively high isosteric heat. Because the Table S1 values were obtained under different temperatures, pressures, gas compositions, activation methods, and selectivity definitions, the entries are not strictly comparable, selectivity least of all.

While the present study focuses on powdered WCB‐derived porous carbons, future development should consider structured formats such as pellets, monoliths, or printed architectures to improve handling, pressure‐drop behavior, and device integration. Evaluation under process conditions, including tolerance to water vapor, regeneration energy in a working cycle, and long‐term cycling stability, will likewise be required to establish post combustion performance. The recent demonstration of 3D‐printed amine‐functionalized activated carbon monoliths for direct air capture [62] illustrates how carbon adsorbents can be engineered into practical capture platforms, providing a useful direction for translating waste‐derived carbons into deployable CO_2_ capture systems.

## 3 Conclusion

This work demonstrates a waste‐valorization strategy for converting waste cigarette butts, a persistent plastic‐rich environmental pollutant, into hierarchical porous carbons for CO_2_ capture. Using Na_2_CO_3_ as a relatively mild activating agent, the pore architecture of whole‐cigarette‐butt‐derived carbon was systematically tuned across the WCB‐*x* series. The results show that CO_2_ adsorption is not maximized simply by increasing activation severity, but by achieving an optimum balance between microporous adsorption domains and mesoporous/macropore‐like transport pathways. Among the activated carbons, WCB‐83 exhibited the best balance of pore architecture, delivering a CO_2_ uptake of approximately 3.0 mmol g^−^^1^ at 273 K and 1 bar. The decline in uptake for WCB‐91 highlights the importance of controlling over‐activation, which can reduce the effective high‐affinity microporous volume through pore widening or structural degradation. Temperature‐dependent adsorption measurements further showed apparent isosteric heats of approximately 53 to 59 kJ mol^−^^1^ for WCB‐83, consistent with strong CO_2_ confinement within narrow micropores and ultra micropores. In addition, WCB‐83 displayed high IAST‐predicted CO_2_/N_2_ selectivity for an ideal dry 15:85 CO_2_/N_2_ mixture, indicating preferential CO_2_ adsorption under post‐combustion‐relevant conditions. These findings establish a structure–performance relationship for carbons derived from cigarette butts and demonstrate how persistent, plastic rich litter can be converted into functional adsorbents for carbon capture. More broadly, the work provides a pathway that links waste remediation with the development of carbon capture materials, while advancing the use of waste‐derived carbon in environmental separation technologies. Deliberate control over pore structure and surface chemistry could enable functionalization strategies that further improve CO_2_ capture capacity, highlighting clear scope to optimize performance beyond the present system. The environmental benefit of this waste‐valorization route remains to be quantified against its Na_2_CO_3_ inputs of up to 10 g per gram of biochar, thermal treatments at 400°C and 800°C, 1 M HNO_3_ treatment, and repeated washing. Further development should prioritize reduced activator consumption, reagent recovery and water reuse, with wash‐stream treatment addressing dissolved salts and any leached contaminants alongside pH adjustment. However, this valorization approach should complement source reduction measures, as preventing cigarette butt pollution remains the preferred environmental outcome.

## 4 Experimental

### 4.1 Materials

Waste cigarette butts were supplied from litter collection activities undertaken by environmental charity, No More Butts. Sodium hydroxide and anhydrous sodium carbonate were purchased from Merck with purity greater than 99%. Absolute ethanol was purchased from Sigma–Aldrich and used as received without further purification. Milli‐Q water was produced using a Milli‐Q purification system with a resistivity of 18.2 MΩ cm at 25°C.

### 4.2 Method

Filter‐only cigarette butt (FCB) precursor was prepared by manually separating the cigarette filters from the collected cigarette butts. The separated filters were washed sequentially with water and ethanol, then dried under vacuum at 80°C overnight. For the whole cigarette butt (WCB) precursor, the entire cigarette butt waste was first separated from visible foreign contaminants, including soil particles, plastics, and other debris. The WCB material was then washed thoroughly with copious amounts of water, typically 5–6 times, until the wash water became clear and the waste appeared free of loosely attached contaminants. The washed WCB precursor was dried under vacuum at 100°C overnight. The dried precursors were initially pyrolyzed under an argon atmosphere at 400°C for 2 h to produce carbonaceous biochar intermediates in an OTF‐1200X tube furnace. The resulting WCB‐derived biochar was then chemically activated using either anhydrous Na_2_CO_3_ or NaOH. For Na_2_CO_3_ activation, the biochar was physically mixed with anhydrous Na_2_CO_3_ at biochar: Na_2_CO_3_ mass ratios of 4:5, 3:5, 2:5, 1:5, and 1:10, corresponding to Na_2_CO_3_ contents of approximately 56, 63, 71, 83, and 91 wt.%, respectively. The mixed samples were then immediately subjected to the second pyrolysis/activation step. For NaOH activation, a biochar/NaOH mass ratio of 1:5 was used. NaOH was dissolved in deionized water before mixing with the biochar to improve contact and promote more uniform distribution of the activating agent. The resulting biochar/NaOH mixture was dried again prior to activation; this drying also provided additional contact time between NaOH and the biochar matrix. Chemical activation was carried out at 800°C for 2 h under flowing argon. After activation, the pyrolyzed products were recovered and washed twice with Milli‐Q water. The samples were then acid‐washed using 1 M HNO_3_ to remove residual inorganic species, followed by further washing with deionized water five times and then with Milli‐Q water three times until the filtrate reached approximately pH 6–7. The final activated carbons were dried under vacuum at 100°C overnight before characterization. The washed, dried product yield was 52%–58% relative to the starting biochar (54% ± 2% for WCB‐83), giving an overall WCB‐83 yield of approximately 17% when combined with the approximately 32% carbonization yield.

### 4.3 Characterization

Scanning electron microscopy (SEM) was performed using an FEI Verios 460 field‐emission scanning electron microscope operated at an accelerating voltage of 3 kV. Fourier‐transform infrared (FTIR) spectra were recorded using a PerkinElmer FTIR spectrometer. Raman spectra were collected using a WITec alpha300 R Raman microscope equipped with a 785 nm laser. Spectra were acquired over the range of 200–3000 cm^−^^1^ with a spectral resolution of 2.7 cm^−^^1^, using a 10× objective. Each measurement was performed at a laser power of 5 mW, with an integration time of 10 s and 20 accumulations. Five replicate measurements were collected for each sample. Thermogravimetric analysis (TGA) was carried out using a Mettler Toledo TGA/DSC 3+ system under nitrogen flow. Samples were heated from 25 to 800°C at a heating rate of 10°C min^−^^1^. Nitrogen adsorption–desorption measurements were conducted at 77 K using a Micromeritics TriStar II surface area and porosity analyzer. Prior to analysis, approximately 100–120 mg of powdered sample was degassed at 373 K for 24 h. Brunauer–Emmett–Teller (BET) surface areas and micropore size distributions were computed from the 77 K N_2_ isotherms using the Tarazona NLDFT model, and ultra‐micropore size distributions from the 273 K CO_2_ isotherms using the CO_2_ DFT model, both within the *MicroActive* application. The CO_2_ kernel treats CO_2_ on carbon in slit pores with a homogeneous surface; distributions were obtained by non‐negative regularization (parameter 0.1), giving a fit standard deviation of 0.118 cm^3^ g^−^^1^ (STP) for WCB‐83. Mesopore size distributions were estimated with the Barrett–Joyner–Halenda (BJH) method. CO_2_ adsorption measurements at 273 K were performed using approximately 200 mg of sample. Before analysis, samples were degassed at 200°C for 24 h and then analyzed using an ice–water bath maintained at 0.6°C ± 0.1°C measured with a thermocouple before and after measurement. Isosteric heats (*Q_st_*) were obtained from the CO_2_ isotherms at 273, 283, and 298 K, with uptake converted to mmol g^−^^1^. At each loading between 0.4 and 1.0 mmol g^−^^1^, the pressure at each temperature was interpolated piecewise linearly in *q* versus ln *P*, and

$$Q_{ST}=-R{\left[\frac{\partial InP}{\partial \left(1/T\right)}\right]}_{q}$$

was obtained by ordinary least squares regression of ln *P* against 1/*T*. CO_2_ adsorption–desorption cycling tests were conducted by repeating the degassing and adsorption measurement for five cycles. For room‐temperature CO_2_ and N_2_ adsorption measurements, samples were degassed under the same conditions, and the adsorption analysis was performed with the sample tube immersed in a water bath (analysis Dewar) maintained at 25°C ± 1°C.

## Author Contributions

**Joseph F. Olorunyomi**: conceptualization, investigation, writing – original draft, writing – review and editing, validation, methodology, software, formal analysis, project administration, data curation, supervision, resources, visualization. **Shinen Yu**: investigation, methodology, writing – review and editing, data curation, validation, project administration. **Lison Shaju**: investigation, writing – review and editing, methodology, data curation, validation, project administration. **Nandish Hosadoddi Srikantamurthy**: investigation, data curation, writing – review and editing, formal analysis, software, methodology. **Jianjin Ruan**: investigation, writing – review and editing, software, formal analysis, data curation, methodology. **Jeremy D. Landry**: investigation, writing – review and editing, software, formal analysis, data curation, methodology. **Xavier Mulet**: writing – review and editing, supervision, resources, conceptualization, validation, funding acquisition. **Ravichandar Babarao**: writing – review and editing, supervision, resources, conceptualization, validation, funding acquisition.

## Conflicts of Interest

The authors declare no conflicts of interest.

## Supporting information




**Supporting File**: gch270171‐sup‐0001‐SuppMat.docx.

## Data Availability

The data that support the findings of this study are available from the supplementary information.
