# Supplementary material for: Pore‐Hierarchy Engineering of Cigarette Butt Waste Into Porous Carbon for CO2 Adsorption
Source: Glob Chall. 2026 Oct 24;10(10):e70171. doi: 10.1002/gch2.70171 (PMC13695153; doi:10.1002/gch2.70171)
Supplement: Supplementary file 1 [file GCH2-10-e70171-s001.docx]

Supplementary Information

**Pore-Hierarchy Engineering of Cigarette Butt Waste into Porous Carbon for CO₂ Adsorption**

*Joseph F. Olorunyomi,** *Shinen Yu,*^†^ *Lison Shaju,*^†^ *Nandish Hosadoddi Srikantamurthy, Jianjin Ruan, Jeremy D. Landry, Xavier Mulet,* Ravichandar Babarao**

J. F. Olorunyomi, S. Yu, L. Shaju, H.S. Nandish, J. Ruan, J.D. Landry, X. Mulet, R. Babarao

School of Science, RMIT University, Melbourne, Victoria 3001, Australia.

Email: [joseph.olorunyomi@rmit.edu.au](mailto:joseph.olorunyomi@rmit.edu.au), [xavier.mulet2@rmit.edu.au](mailto:xavier.mulet2@rmit.edu.au), ravichandar.babarao@rmit.edu.au

R. Babarao

Centre for Advanced Materials & Industrial Chemistry (CAMIC) and ARC Centre of Excellence for Electrochemical Transformation of Carbon Dioxide, School of Science, RMIT University, Melbourne, Victoria 3001, Australia.

Email: ravichandar.babarao@rmit.edu.au

^†^These authors contributed equally to this work

**S1: Thermal behavior of cigarette-butt precursors and activation mixtures**

TGA and DTG analysis of the char–Na_2_CO_3_ mixtures provided insight into the thermal stability and transformation of the mixed char–salt system before post-activation washing. Pristine WCB-derived char underwent progressive mass loss during heating and retained approximately 64% of its initial mass at 800 °C, whereas pure Na_2_CO_3_ remained largely stable, with approximately 96% residual mass as shown in Figure S1b. The char– Na_2_CO_3_ mixtures showed intermediate residual masses that increased with Na_2_CO_3_ content, reflecting the increasing contribution of thermally stable Na_2_CO_3_ to the total residue. Therefore, the solid remaining after thermal treatment should be regarded as a mixture of carbonaceous char, unreacted Na_2_CO_3_, and possible sodium-containing inorganic intermediates, from which the final porous carbon is obtained after washing. The DTG profiles in Figure S1c further indicate that Na_2_CO_3_ modifies the high-temperature transformation of the carbonized char. The low-temperature feature near 90 °C corresponds to removal of adsorbed moisture and weakly bound volatile species, while the feature around 470 °C, most evident in pristine char, is associated with further decomposition of residual labile carbonaceous species. At higher temperatures, pristine char exhibits a mass-loss feature around 678 °C, whereas the corresponding feature in the Na_2_CO_3_-containing mixtures shifts slightly to approximately 660 °C. An additional feature near 740 °C is also observed for the char– Na_2_CO_3_ mixtures but is less pronounced in pristine char. Since pure Na_2_CO_3_ remains largely stable up to 800 °C under the conditions examined, these high-temperature events are likely carbonate-assisted transformation of the mixed char–salt system and also probably interfacial Na_2_CO_3_–char reactions, gasification of reactive carbon sites, formation of Na-containing intermediates, and rearrangement of the carbon matrix. A direct comparison between WCB-83 and the corresponding NaOH-activated sample, WCB-OH-83, further showed that NaOH induces earlier and more extensive char transformation than Na_2_CO_3_ (Figure S1d and e). These profiles indicate differences in the thermal transformation of the char–activator mixtures under the conditions examined.

**Figure S1**. (a) TGA profiles of WCB and FCB precursors showing higher residue formation from WCB at 400 °C. (b) TGA profiles of WCB-derived biochar, pure Na_2_CO_3_ and char– Na_2_CO_3_ mixtures with different Na_2_CO_3_ contents. (c) Corresponding DTG profiles from (b) showing carbonate-assisted high-temperature transformation of the char–salt mixtures. (d,e) Comparison of TGA and DTG profiles for WCB-derived biochar containing 83 wt.% Na_2_CO_3_ or NaOH, showing the stronger and earlier activation behavior of NaOH and (f). TGA profiles of WCB-x samples after activation and removal of Na_2_CO_3_.

**Figure S2**. N_2_ adsorption-desorption isotherms of WCB-83 (S_BET_: 750 m^2^ g^-1^), WCB-83-OH (S_BET_: 900 m^2^ g^-1^) and WCB-0 (S_BET_: 0.6 m^2^ g^-1^) at 77 K.

**Figure S3**. Raman spectra and fitted D and G bands of WCB-83 and WCB-83-OH. Dashed curves represent the fitted peak components, while horizontal arrows indicate the FWHM values of the D and G bands.

**S2. CO₂ Adsorption Performance of WCB-83 versus WCB-OH-83**

The CO₂ adsorption performance of WCB-83 was compared with that of WCB-OH-83 to assess the effect of activation chemistry on adsorption behavior. Although NaOH is a stronger activating agent, WCB-OH-83 exhibited a substantially lower CO₂ uptake of approximately 1.2 mmol g⁻¹ at 273 K and 1 bar, compared with approximately 3.0 mmol g⁻¹ for WCB-83. The non-activated WCB-0 showed only limited CO₂ uptake of approximately 0.5 mmol g⁻¹. These results indicate that stronger chemical activation does not necessarily produce a more effective CO₂ adsorbent. Instead, the Na_2_CO_3_ route appears to generate a more favorable balance of microporosity and pore accessibility. The higher uptake of WCB-83 is therefore attributed to a better-developed hierarchical pore network, in which narrow micropores provide the main CO₂ adsorption sites while larger pores improve access to the internal adsorption domains.

**Figure S4**. CO_2_ adsorption isotherms of WCB-83, WCB-83-OH and WCB-0 at 273 K.

**Figure S5**. Isosteric heat of CO₂ adsorption on WCB-83. (a) Clausius–Clapeyron plots (ln P versus 1/T) at fixed loadings of 0.4 to 1.0 mmol g⁻¹, from the isotherms at 273, 283 and 298 K; lines are linear fits (R² labelled). (b) Isosteric heat Q_st_ versus CO₂ loading from the fitted slopes; error bars are the regression standard error.

**Figure S6**. CO₂ adsorption cycling stability of WCB-83 at 273 K. (a) CO₂ adsorption isotherms over five consecutive adsorption–desorption cycles. (b) CO₂ uptake at 1 bar for each cycle, showing stable capacity retention and adsorption reversibility.

**S3. Ideal adsorbed solution theory calculation**

Single-component CO₂ and N₂ adsorption isotherms measured at 298 K were fitted with the Toth model, which was chosen because it captures the energetic heterogeneity of the adsorption sites expected in a hierarchical porous carbon that combines microporous adsorption domains with larger transport pores. The fitted isotherms were then used as input for Ideal Adsorbed Solution Theory (IAST) calculations, performed with the *pyIAST* package in Python (Google Colab), to predict the CO₂/N₂ adsorption selectivity of a dry 15:85 mixture. The fitted equation was:

$$q=\frac{n_{m}KP}{\left[ 1+(KP)^{t} \right]^{1/t}}$$

where $q$is the adsorbed amount, $n_{m}$is the saturation capacity, $K$is the affinity constant, $P$is the absolute pressure, and $t$is the heterogeneity parameter. The fitted Toth parameters were $n_{m}=10.0000\mathrm{mmol}g^{-1}$, $K=0.3427\mathrm{ba}r^{-1}$, and $t=0.3665$for CO₂, and $n_{m}=0.4243\mathrm{mmol}g^{-1}$, $K=0.0581\mathrm{ba}r^{-1}$, and $t=1.0000$for N₂. The higher $K$ value for CO₂ indicates stronger adsorption affinity relative to N₂, while the lower CO₂ $t$value reflects adsorption heterogeneity within the porous carbon framework.

IAST calculations were performed for an ideal dry binary mixture containing 15% CO₂ and 85% N₂ over a total pressure range of 0.02–1.0 bar. The CO₂/N₂ selectivity was calculated as:

$$S_{CO_{2}/N_{2}}=\frac{x_{CO_{2}}/y_{CO_{2}}}{x_{N_{2}}/y_{N_{2}}}$$

where $x_{i}$and $y_{i}$are the adsorbed-phase and gas-phase mole fractions, respectively. Because the fitted CO₂ $n_{m}$ value reached the imposed upper fitting bound, the Toth model was used primarily to provide a smooth representation of the measured isotherm for IAST interpolation within the experimental pressure range, rather than to assign a definitive physical saturation capacity.

**Figure S7**. Toth versus single site Langmuir fits for WCB-83 at 298 K: (a) measured CO₂ isotherm with both fits (RMSE inset); (b) CO₂ fit residuals; (c) measured N₂ isotherm and fit (Toth exponent t = 1, so the fits coincide); (d) IAST CO₂/N₂ selectivity for a dry 15:85 mixture versus total pressure.

**Table S1**. CO₂ adsorption properties of WCB-83 and selected porous carbons and other adsorbents reported in the literature.

| **Material** | **CO_2_ uptake at 273K, 1 Bar (mmol/g)** | **Ultra micropore size (< 1 nm)** | **IAST CO_2_/N_2_ Selectivity (298K)** | ***Q_st_* (kJ/mol)** | Ref |
| --- | --- | --- | --- | --- | --- |
| Biomass-tar-derived carbon | 5.03 | 0.8 | 19.7 | 48.5 | ^[1]^ |
| UFK-600-0.2 | 4.03 | ~1 | 35 | 53 | ^[2]^ |
| Hawaii nutshell carbon (HNSC-700-3) | 7.07 | ~1 (1.1) | 20 (10:90) | 24-39 | ^[3]^ |
| Potassium sodium tartrate self-activated carbon (PSTC-650) | 4.99 | ~1 | 20 | 22–40 | ^[4]^ |
| NOPC1 | 3.15 | 0.8 | 16.9 | 23.6 | ^[5]^ |
| Boron-doped water chestnut shell carbon (WSCB-700-3) | 7.01 | ~1 | 18 (10:90) | 18–31 | ^[6]^ |
| MR-1-500 | 6.24 | NR | 52.9 | 37 | ^[7]^ |
| N,S-codoped coconut shell carbon (CSCT-700-2) | 6.46 | ~1 | 17 | 22-37 | ^[8]^ |
| Mg-MOF-74 | 10.22 | NR | NR | 42 | ^[9]^ |
| NPC-600 | 4.77 | 0.9 | NR | 57 | ^[10]^ |
| PUF-400-KOH-1-700 | 6.67 | 0.7/0.86 | NR | 63 | ^[11]^ |
| WCB-83 | 3.0 | 0.56 | ~110 (Toth) / ~80 (Langmuir) | 53-59 | This work |

NR = not reported. Literature values provide context; direct comparisons require matched temperature, pressure and, for IAST selectivity, gas composition.

## References

[1]	H. Yuan, J. Chen, D. Li, H. Chen, Y. Chen, *Chemical Engineering Journal* **2019**, 373, 171.

[2]	Q. Yu, J. Bai, J. Huang, M. Demir, B. N. Altay, X. Hu, L. Wang, *Molecules* **2022**, 27, 6816.

[3]	X. Liu, Y. Wang, Z. Chen, X. Hu, Q. Xiao, L. Zhao, M. Demir, M. Kilic, M. K. Al Mesfer, L. Wang, *Biomass and Bioenergy* **2026**, 214, 109531.

[4]	M. Che, Y. Yin, X. Liu, Y. Liu, Q. Xiao, L. Zhao, M. Demir, O. C. Altinci, L. Wang, X. Hu, *Fuel* **2027**, 428, 140503.

[5]	C. Guo, Y. Sun, H. Ren, B. Wang, X. Tong, X. Wang, Y. Niu, J. Wu, *Energies* **2023**, 16, 5222.

[6]	J. Wang, Y. Yin, X. Liu, Y. Liu, Q. Xiao, L. Zhao, M. Demir, U. B. Simsek, L. Wang, X. Hu, *Langmuir* **2026**, 42, 6167.

[7]	H. Cong, M. Zhang, Y. Chen, K. Chen, Y. Hao, Y. Zhao, L. Feng, *Carbon* **2015**, 92, 297.

[8]	J. Shao, Y. Wang, M. Che, Q. Xiao, M. Demir, M. K. Al Mesfer, L. Wang, X. Hu, Y. Liu, *Journal of the Energy Institute* **2025**, 123, 102273.

[9]	D.-A. Yang, H.-Y. Cho, J. Kim, S.-T. Yang, W.-S. Ahn, *Energy Environ. Sci.* **2012**, 5, 6465.

[10]	J. Wang, I. Senkovska, M. Oschatz, M. R. Lohe, L. Borchardt, A. Heerwig, Q. Liu, S. Kaskel, *Journal of Materials Chemistry A* **2013**, 1, 10951.

[11]	C. Ge, J. Song, Z. Qin, J. Wang, W. Fan, *ACS Applied Materials & Interfaces* **2016**, 8, 18849.
